# Gut–Brain Axis and Brain Microbiome Interactions from a Medical Perspective

**DOI:** 10.3390/brainsci15020167

**Published:** 2025-02-08

**Authors:** Borros Arneth

**Affiliations:** Institute of Laboratory Medicine and Pathobiochemistry, Molecular Diagnostics, University Hospital of the University of Marburg UKGM, Philipps University Marburg, Baldingerstr., 35043 Marburg, Germany; borros.arneth@staff.uni-marburg.de

**Keywords:** gut–brain axis, gut microbiome, brain microbiome, central nervous system, immune system, mechanistic pathways, vagus nerve

## Abstract

**Background:** The gut microbiome directly impacts brain health and activity, meaning the two are closely associated. This relationship suggests a link between microbial imbalances and diseases such as Alzheimer’s, although multiple other contributing factors, such as genetics, also play a part. Additionally, recent studies discovered that cerebrospinal fluid has some microbial deoxyribonucleic acid (DNA), which can be interpreted to mean a microbiome exists in the brain too. The vagus nerve and the central nervous and immune systems are responsible for the connection between the brain and gut microbiome. **Aims and Objectives**: The main aim of this systematic review is to analyze existing research on the gut–brain axis and the brain microbiome to fill the current knowledge gap. **Materials and Methods**: A search was conducted on the PubMed database based on a set of predefined MeSH terms. **Results**: After the search, 2716 articles meeting the MeSH parameters were found in PubMed. This list was then downloaded and analyzed according to the inclusion/exclusion criteria, and 63 relevant papers were selected. **Discussion**: Bacteria in the gut microbiome produce some substances that are considered neuroactive. These compounds can directly or indirectly affect brain function through the gut–brain axis. However, various knowledge gaps on the mechanisms involved in this connection need to be addressed first.

## 1. Introduction

### 1.1. Background

Microbes have evolved over the past decades and influence human gut health, impacting the immune and central nervous system functions. The bacteria, fungi, viruses, and other gut microorganisms symbiotically relate with the host to consume the required nutrients and secret beneficial chemicals for the biochemical processes. The microbiomes consume their needed gut nutrients and secrete chemicals such as amino acids, which manage and control biochemical processes such as immune response and homeostasis [1,2,3,4]. It develops in one’s system from infancy and becomes a crucial part of the metabolic and immune systems. The interdependent relationship suggests a strong relationship between the gut microbiome, cognitive health, and central nervous functions.

Multiple communication pathways link the gut microbiome to the central nervous system (CNS). The gut and the brain communicate through the circulatory system, immune system, vagus nerve, neuroendocrine system, and enteric nervous system (ENS) [4,5,6]. The microbiome produces bloodstream chemicals that easily reach the brain and produce neurotransmitters and metabolites that convey bidirectional information between the gut and the brain. Communications via the immune system occur through cytokines produced in the gut and can travel to the brain through the bloodstream. Communication through the vagus nerve occurs when sensory information from the gut is transmitted to the brain through the nerve fibers, while the brain responds through gut functions. Communications through the neuroendocrine system also occur through the vagus nerve; specialized gut wall cell linings release hormones that travel through the bloodstream, ensuring communications between the two systems. The communication relationships and interdependencies allude to the possibility of gut microbiome contributing to certain neurological disorders, such as Alzheimer’s and multiple sclerosis, alongside other genetic, environmental, and lifestyle influences.

Debates have also risen over the existence of microbes in the brain. The studies hypothesize that bacteria, fungi, and viruses exist in the brain. Possibilities emerged of brain microbiomes that influence the development of neurodegenerative diseases, specifically Alzheimer’s disease (AD) and Parkinson’s disease (PD) [6]. The gut–brain axis is based on the concept of a brain microbiome. According to this theory, the CNS contains a microbiota that has a similar relationship with the human body as that of the gut microbiome [7]. Researchers have also detected disease-associated altered microbiomes related to multiple disorders spanning Alzheimer’s disease, autism spectrum disorder, attention-deficit hyperactivity disorder, Huntington’s disease, major depressive disorder, multiple sclerosis, and Parkinson’s disease [8,9]. Research stipulates that the microbes interact with the CNS to influence neuron signaling, neuroinflammation, and other brain health aspects. Certain studies also found no microbiomes in the brains of AD and PD patients [10]. The lack of consistent information on whether microbes exist in the brain and their relative impacts necessitates further research to bridge the knowledge gap.

The relationship between the gut microbiome and the central nervous system is integral to the future of neurological health. Past studies show that the gut microbiome regulates the immune system, metabolism, and neurotransmitter production; however, the mechanism defining how the microbiomes influence brain functions has not been sufficiently studied. Researchers have found communication pathways such as the circulatory system, immune signaling, and the vagus nerve that they believe, if disrupted, could influence the occurrence of neurological disorders. A plethora of research has been conducted in this field, but a major gap exists in how specific microbes interact with neural processes to influence cognitive health. Further research could reveal the mechanistic gut–brain interaction pathways to determine therapeutic targets when treating neurodegenerative and psychiatric disorders.

### 1.2. Knowledge Gap

Although recent research has shown that the gut microbiome has an influence on the CNS via the gut-brain axis, there is limited knowledge of the mechanisms and pathways involved in the process. The concept of the brain microbiome, which is believed to play a crucial part in the relationship between the gut and the brain, is also new, meaning the amount of research on the subject is limited. These research gaps should be addressed in future research. This systematic review consolidates current knowledge on the topic to highlight these gaps.

### 1.3. Aims and Objectives

- To synthesize existing evidence on the gut–brain axis and investigate the mechanistic pathways that link the gut microbiome and the CNS.
- To explore the novel concept of the brain microbiome and determine its existence and the role it plays in one’s neurological health.
- To identify and analyze existing data on the interactions between the gut and brain microbiomes to determine their collective impact on neurological health factors such as neuroinflammation and neurodegeneration.
- To point out existing gaps in research on the gut–brain axis, especially the mostly unexplored brain microbiome.

## 2. Materials and Methods

The materials and methods section delves into the procedures for finding articles for the systematic review. Systematic reviews follow Preferred Reporting Items for Systematic Reviews and Meta-Analyses (PRISMA) to select and analyze past studies. The PRISMA approach was developed to raise the findings’ transparency, clarity, value, and quality. This section covers the search strategy, inclusion/exclusion criteria, data extraction, data synthesis, quality assessment, and overview of the selected studies.

### 2.1. Search Strategy

This systematic review assesses current studies related to the gut–brain axis. The primary search is performed on the PubMed and Google Scholar databases following the Preferred Reporting Items for Systematic Reviews and Meta-Analyses (PRISMA) technique. The following MeSH terms helped to find the desired articles:

*{“Gut-Brain Axis”/“Brain-Gut Axis”/Gut Microbiota”/“Gastrointestinal Microbiome”} + {“Microbiome”/“Microbial Interactions”/“Microbial Metabolites”/“Dysbiosis”/“Short-chain fatty acids”} + {“Central Nervous System”/“Neuroimmunology”/“Neuroinflammation”/“Blood-Brain Barrier”/“Neurotransmitters”} + {“Neurological disorders”/“Alzheimer Disease”/“Parkinson Disease”/Autism Spectrum Disorder”/“Mental Disorders”} +{“Pathogenesis”/“Disease Susceptibility”/“Risk Factors”/“Gene-environment Interaction”} + {“Genome-wide association study”/“Epigenomics”/“Transcriptome”/“Proteomics”/ “DNA Methylation”} + {“Systematic Review”/“Meta-analysis”/Case-control studies”/“Cohort Studies”}*

The MeSH terms stem from key topics and factors discussed under the gut–brain axis, the gut microbiome, and the brain microbiome. The researcher copied and pasted the code into the PubMed and Google Scholar search bars to reveal a list of related studies. The researcher refined the results by adjusting the search bars’ publication dates, study type, and other components. The results were then exported to an Excel sheet for further analysis.

### 2.2. Inclusion/Exclusion Criteria

Vast research has examined gut–brain axis interactions through distinct perspectives. The inclusion and exclusion criteria guide the researcher in achieving the desired efficacy The inclusion and exclusion criteria hinge on the study type, study focus, population, publication date, language, data availability, and intervention studies. Below is an in-depth perspective of the inclusion and exclusion criteria.

On the study type, the systematic review only includes original research papers, meta-analyses, and systematic reviews. The researcher may also list longitudinal and cross-sectional studies if they are relevant. The prospective journal article topics should focus on the gut–brain axis, brain microbiome, and the gut microbiome. The study populations were human subjects or related animals, such as mice. The publication date of the articles must be between 2015 and 2025 to ensure up-to-date information. The research papers must be in English since it is widely used in scientific research around the globe and would permit peer reviews worldwide. The prospective studies must have original data from reputable sources such as healthcare facilities. Finally, the intervention studies should prioritize brain microbiome over other past topics to ascertain the reliability of the information. The researcher will exclude studies with insufficient data or not specific to the research context.

### 2.3. Study Selection and Quality Assessment

The researcher followed the PRISMA guidelines to select the ideal articles. Two independent reviewers will examine and screen the selected articles; disagreements will be resolved through discussion or a third-party arbitrator The articles will undergo initial screening, full-text analysis, and final analysis and selection. The initial screening focuses on the title and abstract, eliminating any paper irrelevant to the research subject. The full-text analysis examines whether the articles comprise key components such as background information, materials and methods, key findings, discussions, and conclusions. The final screening and selection hinge on the article’s contribution to the subject and determine its inclusion in the paper. The researcher used the Newcastle–Ottawa Scale (NOS) to examine the quality of observational studies.

### 2.4. Data Extraction

Data were extracted from the selected articles to determine their key findings and research outcomes. The main parts covered will include the following:

- Study characteristics: Basic information such as author details, publication name and data, and the research design recorded in a spreadsheet. This information was used to confirm whether the study met all inclusion criteria before inclusion.
- Gut–brain microbiome data: All relevant findings related to the gut–brain axis and microbiomes were extracted and recorded. This information formulated the discussion topics of the systematic review.
- Study limitations: All recorded or observed weaknesses were recorded. This information paints a clearer picture of the current state of research on subjects such as the brain microbiome and highlights what needs to be covered in future research.
- Research outcomes: The main conclusions of each article were noted and summarized before being transferred to the systematic review’s discussion section.

### 2.5. Data Synthesis

All data from this systematic review were recorded and analyzed qualitatively and quantitatively in Microsoft Excel. This approach allowed us to map the relationships between the gut and brain microbiomes and their influence on neurological health. Correlational analyses revealed the underlying trends and potential research gaps.

### 2.6. Quality Assessment

Each selected article was subjected to quality assessment to gauge the impact of its contents on this systematic review. In addition to using the inclusion/exclusion criteria and data extraction processes, documented techniques such as the Newcastle–Ottawa Scale (NOS) and the AMSTAR-2 were used to analyze observational studies and meta-analyses. The quality of a given article was estimated based on the research procedure, particularly the participant selection procedures, the type of data collected, and the comparison of the research outcomes to their aims and objectives.

## 3. Results

### Overview of Selected Studies and PRISMA Diagram

**Figure 1:**
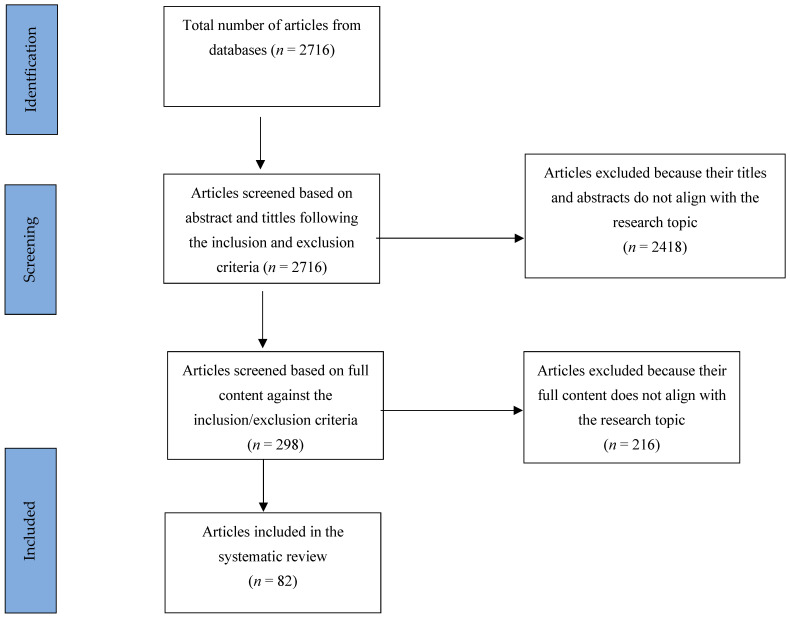
Prisma diagram.

The literature search on the Google Scholar and Pubmed databases yielded 2716 articles. The articles were screened to eliminate the duplicates, resulting in 2237. The titles and abstracts of the remaining articles were then screened to determine conformity to the preferred key terms, gut microbiome and gut–brain axis, leaving *n* = 298 articles. The remaining articles were further screened to remove those lacking data and full texts; in the process, 216 articles were eliminated, leaving 82 journal articles to proceed to the next stage of the research, as shown in the PRISMA diagram (Figure 1).

## 4. Key Findings

The results offer significant insights into the relationship between the gut microbiome and brain health. The findings reveal diverse themes that could offer insights into the research and directions for proper and effective interventions. The preliminary analysis revealed that future research could exploit the relationship between the gut microbiome and brain health to address chronic conditions such as PD and AD. The key themes emerging in the study comprise the association between gut diseases and neurogenerative diseases, gut microbiota and cognitive health, gut microbiota and mental health disorders, gut microbiota and autoimmune diseases, and gut microbiota and cancer risk. Below is an in-depth perspective of the themes.

### 4.1. The Neurological Relevance of the Gut Microbiota

One of the major themes is the neurological relevance of the gut microbiota. The gut microbiome produces neurotransmitters such as serotonin and dopamine that regulate emotional responses, mood, and levels of cognition [8,9,10,11,12]. The neurotransmitters come with neuroactive properties, and therefore, any imbalance in the gut microbiota affects brain function and may cause symptoms such as anxiety, cognitive impairment, and depression [13,14]. The gut–brain axis facilitates the interaction and ensures bidirectional communications between the gut and the neurological system. The above studies prove that the gut microbiome can influence a person’s mental health. The revelation of the link between anxiety, depression, stress disorders, and gut health paves the way for possibilities to exploit future interactions to understand the relationship between the variables. The above themes reveal how microbiome-based properties could help develop therapeutic interventions to address neurological and psychiatric disorders by influencing the gut microbial composition.

### 4.2. Gut Imbalances and Neurological Health

The study also reveals how gut imbalances, such as microbial dysbiosis, can manifest in mental health issues like anxiety in patients; this goes to show the importance of the gut microbiome [15,16,17]. The types and number of microbiomes in the body may cause disease if not properly managed. The studies reveal that certain dietary items, for instance, proteins or sulfur, can imbalance the gut microbiome and predispose the individual to neurological diseases. The studies also revealed that stress contributes to gut imbalances by changing the microbes in the skin and other body parts. Other factors such as aging, drugs, and disease may also alter the nature and amount of microbiome in the gut. Understanding the imbalances and how to address them paves the way for strategic interventions to address each causative factor. The studies attribute gut dysbiosis to neuroinflammation, suggesting that microbial imbalances may contribute to the development of some neurological diseases, especially when combined with other factors such as genetics and the environment [18,19]. The above findings prove that researchers can maintain the gut microbiota balance to promote neurological health, emphasizing the need for a proper diet, lifestyle habits, and strategic therapeutic interventions to balance the gut microbiome and prevent neuroinflammatory disorders.

The gut imbalances stem from dysregulations that affect gut permeability, encouraging inflammatory cytokines to get into the neurological system and trigger conditions such as Alzheimer’s and Parkinson’s diseases [20,21,22]. The current theme sets the pace for exploring gut-based therapies such as probiotics and prebiotics alongside other interventions to restore microbial balance. Other recent studies on animal models under controlled modulations of the gut microbiome revealed that introducing the gut bacteria lowered neuroinflammation, thereby improving mood and general behavior [23,24,25]. This evidence reveals the possibility of treating neurological issues through interventions that target the microbiome. Comprehensive clinical trials could help advance research on the subject and pave the way for practical solutions to gut-based health conditions.

### 4.3. Diseases Where the Gut Microbiota Could Be Pathogenic

The findings also revealed some diseases that could arise from gut microbiota imbalances. The cases where the gut microbiome emerged pathogenic include multiple sclerosis (MS), Parkinson’s disease, and Alzheimer’s disease. MS, characterized by CNS inflammation, has been linked to gut dysbiosis [26]. Gut microbiota contains many metabolites with immunomodulatory characteristics that may trigger pro- or anti-inflammatory responses as they interact with immune cells [27]. For instance, metabolites based on the Trp amino acid, such as indole and kynurenine, are pro-inflammatory, meaning they may trigger the onset of MS. At the same time, the gut microbiome may release anti-inflammatory short-chain fatty acids (SCFAs). The balance between these classes of metabolites determines the pathogenesis of MS in an individual [28].

The results also showed the possibility of Parkinson’s disease being pathogenic [29]. The reduced beneficial bacteria due to age and diet related to dysbiosis trigger Parkinson’s disease symptoms. Increased concentration of harmful bacteria also increases alpha-synuclein clumping, which forms Lewy body aggregations that may infiltrate the neurological system and affect some pathways [30]. Gut dysbiosis further raised intestinal permeability, known to increase neuroinflammation and oxidative stress, both of which directly contribute to Parkinson’s disease.

The gut microbiome also plays a pathogenic role in Alzheimer’s Disease progression. The studies show that bacteria in gut microbiota secret amyloids and lipopolysaccharides in large quantities, which modulate signaling pathways and accelerate the production of pro-inflammatory cytokines linked to the pathogenesis of Alzheimer’s Disease [31]. Variations in gut permeability could also destabilize the blood–brain barrier leading to metabolite imbalances and neuroinflammation. For instance, the release of SCFAs, which are anti-inflammatory in nature, could drop due to gut dysbiosis and increase the risk of Alzheimer’s disease [32].

### 4.4. Diseases Where Modulation of the Gut Microbiota Could Be Beneficial

The findings also showed that modulating the gut microbiota could alleviate certain disease symptoms. It emerged that among patients with autism spectrum disorder (ASD), depression, and anxiety, effective management of the gut microbiome could resolve the symptoms leading to quick recovery. For instance, healthcare practitioners can address ASD by improving the gastrointestinal and behavioral symptoms through techniques such as fecal transplantation, probiotics, breastfeeding and formulas, gluten- and casein-free (GFCF) diets, ketogenic therapy, and others [33]. Some compounds produced by gut bacteria are bioactive or neuroactive, and can impact brain function in Alzheimer’s patients. The research showed that people with the disease have a different gut microbiome compared to others. Relatively, dysregulating the immune system due to gut dysbiosis may trigger the body to form autoantibodies in ASD patients, which could reduce hypersensitive responses and improve one’s behavioral symptoms [34].

The gut microbiome resolves depressive symptoms by producing neurotransmitters such as serotonin, which regulate an individual’s mood after crossing the gut–brain axis [35,36]. It reduces inflammation levels in the signaling pathways in the vagus nerve to improve the person’s mental well-being. The gut bacteria also metabolizes tryptophan, an amino acid that promotes the secretion of serotonin [37]. This finding justifies the use of the gut microbiota to treat depression. The gut microbiota’s production of serotonin and other neurotransmitters further manages mood and stress response [38]. The hormones improve the ability of the vagus nerve to communicate and regulate mood. Balancing the gut microbiome promotes a healthy immune system, needed to minimize neuroinflammation and subsequent anxiety and stress. Other studies showed that some gut bacteria can secrete cortisol, one of the body’s key hormones for stress control.

Thus, whereas gut microbiome imbalance could influence pathogenesis of various diseases such as MS, Alzheimer’s, and Parkinson’s, it can also benefit conditions such as ASD, depression, and anxiety. Knowledge of the complex interactions between gut microbiota and neurological health is important to prevent the conditions and develop effective therapies to address their symptoms. Table 1 summarizes the relationships between the gut microbiota and human neurological health.

### 4.5. Mechanistic Pathways Involved in the Gut–Brain Axis

Multiple pathways, such as the vagus nerve and the immune system, link the brain to the gut and facilitate distinct communications. The systems facilitate gut–brain axis communication in either direction [39,40,41,42,43]. In fact, most studies showed that treatment and therapies on the vagus nerve can lower depression symptoms and other similar conditions [44,45]. The gut and CNS connect through metabolites released because of microbial bacteria, such as SCFAs [46]. Some of the SCFAs include propionate, butyrate, and acetate, and help to control neuroinflammation through the blood–brain barrier (BBB) [47]. The gut bacteria further produce indole-based derivatives, which regulate serotonin pathways that modulate a person’s mood and sleep [48]. The primary function of the immune system in this process is to act as a route of communication between the gut and the brain through cytokine and microglial activity. The pathways work together to create the gut–brain axis.

### 4.6. Emerging Evidence of the Brain Microbiome

Despite scientists discovering the link between the gut microbiome and the neurological system decades ago, the concept of a separate brain microbiome only emerged recently [49,50,51,52]. Advanced sequencing technologies on brain tissue revealed traces of microbial DNA, implying that some microbial colonies exist within the system. Some studies reveal that the microbes may enter the brain through the circulatory or lymphatic systems, potentially when the BBB is disrupted. The gap in the findings necessitates further research to confirm the mechanisms involved and the relative implications for cognitive health [53,54].

The data also showed that the brain microbiome acts similarly to the gut microbiome to influence neuron signaling, neuroinflammation, and synaptic plasticity [41,55,56]. For instance, cerebrospinal fluid has microbial byproducts that affect brain function [57,58]. The concept is, however, new and lacks sufficient research to justify its use. Further studies could help explore the subject in depth and offer insights and improvements.

### 4.7. Interplay Between the Gut and Brain Microbiomes

The gut–brain axis, where the gut and brain microbiomes interact, influences a person’s neurological health through multiple pathways [59,60,61]. For instance, the gut microbiome releases B cells, which move to the CNS and link to conditions such as multiple sclerosis [42,62]. Notably, SCFAs and other metabolites from the gut microbiome impact brain function, suggesting that they are secreted in the brain microbiome [63]. Advanced research shows that the gut–brain axis is a critical factor in autism [64,65]. Healthcare practitioners could adopt microbial interventions to reduce the its neurological symptoms and boost a person’s gastrointestinal health [66,67]. The results necessitate examining the gut and brain microbiome as a unified microbial system with the same function rather than two separate entities.

### 4.8. Challenges and Research Gaps

The positive progress in the research on gut–brain axis studies over the years has been marred with challenges and gaps, especially in healthcare settings. For instance, fecal microbiota transplantation (FMT) involves transferring fecal matter from one person to another, which is potentially unsafe. Another problem is that most studies rely on animal models, making their findings inapplicable to humans and could be misinterpreted.

A significant knowledge gap in the systematic review is the scanty research on the pathways and mechanisms involved in the gut–brain axis, especially communication between the gut microbiome and the brain. A few studies discuss the individual microbial species and metabolites that participate in the process, albeit unclearly. Further research, especially longitudinal studies, is necessary to explore and understand the pathways. Table 2 summarizes the most important findings.

### 4.9. Most Significant Findings

Table 2 summarizes the key findings from this systematic review.

## 5. Discussion

The gut microbiome significantly contributes to neurological health. The gut bacteria influence an individual’s mood, overall mental health, and even the pathogenesis of various disorders such as Parkinson’s disease. The links highlight the importance of the gut–brain axis and communication between the gut and the nervous system. The studies also attribute some conditions to imbalances in the gut microbiota. For instance, depression and anxiety are linked to gut dysbiosis, which may affect gut permeability and contribute to neuroinflammation. These effects are attributed to changes in gut microbial stability. For instance, changes in permeability cause bacterial amyloids, lipopolysaccharides, and cytokines to cross the blood–brain barrier into the neurological system and affect various pathways, thus causing symptoms related to Alzheimer’s, Parkinson’s, and other diseases.

Emerging evidence also suggests a strong relationship between nutrition, the gut microbiome, and brain function [68,69,70,71]. A healthy diet encourages the formation of a diverse gut microbiome whose bacteria produces valuable compounds such as SCFAs in large quantities. Thus, a healthy diet indirectly supports brain development and function, while a poor diet increases the risk of gut dysbiosis linked to neuroinflammation and conditions such as Alzheimer’s disease [72]. Scientists and physicians recognize the therapeutic opportunity the gut–brain axis offers to explore practical interventions and practices, especially for diseases such as Alzheimer’s and Parkinson’s [69,73,74,75].

The connection through systems such as the vagus nerve means these pathways can be targeted in such interventions. Some clinical trials have shown that adding new strains to a patient’s gut microbiome could strengthen it and improve their cognition [68,76]. These bacteria include bifidobacterium, lactobacillus, and others [77,78,79,80]. This information can be used to develop effective treatments such as FMT for brain-related disorders [40,81,82]. However, safety concerns limit its applicability, necessitating standard FMT protocols and additional research on how microbiota transfer.

### 5.1. Study Limitations

Certain limitations influenced the study outcomes and could affect the strength and quality of the findings. The limitations border reliance on correlation data, variability of study designs, non-standardized microbiome analysis technique, minimal understanding of the mechanistic pathways, little clinical evidence on microbiome-based therapies, and the emerging but inconclusive research on the brain microbiome. Most reviewed studies relied on correlational data to understand the relationship between gut microbiota and neurological health, avoiding the causal relationship. The minimal longitudinal studies limit the knowledge of whether the imbalances in the gut microbiota directly contribute to the consequences or emerge as a result of the conditions. The research also varied the study designs, limiting the generalization of the findings across diverse disease and demographic conditions. The non-standardized microbiome analysis technique limited the ability to compare findings across distinct research due to varying sample collection methods, data interpretation, and depth sequencing. Little knowledge of the mechanistic pathways limits information on how specific microbial species and metabolic pathways influence brain function, limiting the outcome. The limited clinical evidence on microbiome-based therapies undermines the knowledge of their efficiency and requires more research to reveal their efficacy. Finally, the emerging and inconclusive research on brain microbiomes is inconclusive, inconsistent, and controversial. Advanced research would offer insights into the matter.

### 5.2. Study Implications

The study findings significantly imply practice in neuroscience, mental health, and clinical practice. The revelation of how gut microbiota influences neurological function increases the evidence on the subject and could contribute to future studies. Thus, the key implications include the following:

- *Advancements in Neurological and Psychiatric Research*—This research reveals how gut microbiota influences neuroinflammation, neurotransmitter regulation, and cognitive function and presents new perspective to handle neurological diseases such as Alzheimer’s, Parkinson’s, and multiple sclerosis
- *Potential for Microbiome-Based Therapies*—The results reveal the importance of probiotics, prebiotics, and fecal microbiota transplantation to address the neurodegenerative and psychiatric disorders
- *Public Health and Preventive Strategies*—The relationship between gut microbiota and neurological health reveals the need for a proper diet, lifestyle, and environmental factors to prevent neurological diseases. Public health policies could improve the preventive measures to address the issues.
- *Clinical and Diagnostics Innovations*—The gut bacteria composition could be a biomarker for detecting early neurodegenerative and psychiatric disorders, permitting earlier interventions and proper disease management.
- *Interdisciplinary Collaboration*—The findings reveal the need for neuroscientists, microbiologists, and clinicians to collaborate and establish a comprehensive knowledge of the gut–brain axis and propose strategic interventions.

### 5.3. Study Recommendations

The findings of this study point to a few recommendations that could improve research, clinical practice, and public health strategies to mitigate issues with the gut–brain axis and neurological health. The following recommendations could apply to the current study:

- *Future research directions*—The findings provide direction for future longitudinal and causal studies, mechanistic investigations, and brain microbiome validation.
- *Clinical and Therapeutic Applications*—The findings could help establish the development of microbiome-based therapies, personalized medicine approaches, and early diagnosis and biomarker identification.
- *Public Health and Lifestyle Recommendations*—Healthcare stakeholders should adopt effective stress and lifestyle management, microbiome-friendly diets, and education and awareness campaigns.
- *Interdisciplinary Collaboration*—Interdisciplinary teams could collaborate to integrate neuroscience and microbiome research and policy development for gut health interventions.

## 6. Conclusions

This systematic review reveals the role of gut microbiota in neurological health. It influences neurodegenerative diseases, cognitive functions, mental health disorders, autoimmune diseases, and cancer risks. The results reveal a strong gut–brain connection in which microbial imbalances cause neuroinflammation, neurotransmitter dysregulation, and disease progression. The studies demonstrate that altering gut microbiota triggers conditions such as Alzheimer’s, Parkinson’s, depression, and schizophrenia, revealing the need for microbiome health. Significant progress has been made on the gut–brain axis; however, major gaps exist in the causal relationships, the microbial mechanisms, and the standardized microbiome diagnostic tools. Researchers could conduct further trials to validate the long-term effectiveness of the interventions. The study implications border clinical practice, public health strategies, and therapeutic advancements. Subsequent research should focus on longitudinal research, standardized microbiome practice, and interdisciplinary collaborations. The recommended strategies would streamline the treatment of neurological and psychiatric health issues to improve population health.

## Figures and Tables

**Table 1:** Diseases linked to gut microbiota.

Disease/Disor.	Role(s) Played by Gut Microbiota	Potential Interventions	Risk Factors	Mechanisms
Multiple Sclerosis	Pathogenic: Imbalances in the gut microbiome may trigger autoimmunity, which could lead to MS symptoms [21,26,27].	Probiotics and dietary adjustments to reduce gut imbalances and dysbiosis.	High-fat diets, genetics, antibiotic usage	The gut microbiota influences immune system regulation which may trigger autoimmune responses that attack the CNS [28].
Parkinson’s Disease	Pathogenic: Gut microbiota may promote protein aggregation and affect neurological pathways related to Parkinson’s disease [29,30].	Probiotics and supplementation with SCFAs to improve microbial balance.	Aging, pesticide exposure, poor diet	Microbial imbalance is linked to the accumulation of alpha-synuclein, which is associated with neurodegeneration and motor dysfunction [39].
Alzheimer’s Disease	Pathogenic: Gut dysbiosis increases the risk of neurological inflammation, which could impact Alzheimer’s markers [31,32].	Probiotics and anti-inflammatory diets to reduce neuroinflammation and subsequent Alzheimer’s symptoms.	Aging, high-fat diets, chronic inflammation of neurological pathways due to external factors	Dysbiosis may activate neuroinflammatory pathways and increase the concentration of beta-amyloid plaques which is linked to cognitive decline [32,40].
Autism Spectrum Disorder	Beneficial: Changes in the gut microbiome can positively impact one’s behavior [17,33].	Probiotics and other dietary interventions to improve gut health.	Overdependence on formula feeding (among infants) as opposed to natural foods and breastfeeding, risks associated with cesarean delivery	Through the gut–brain axis, gut microbiota may affect a person’s behavior and neurodevelopment [41].
Depression	Beneficial: The gut microbiome’s contents can help reduce mood dysregulation [9,13].	Synbiotics and probiotics to minimize gut imbalance.	Stress, poor diet, antibiotic usage	Signals from the gut may increase serotonin production and modulate the hypothalamic–pituitary–adrenal (HPA) axis, improving mood [34].
Anxiety	Beneficial: Improvements in gut permeability could reduce anxiety-related symptoms [13,14].	Probiotics and fecal microbiota transplantation (FMT) to improve the gut microbiome’s health. Introducing bacteria from a healthy person’s gut could help restore balance, but the patient’s diet will play an important part.	Chronic stress, poor diet	The gut microbiome may induce immune modulation and neurotransmitter production [42].

**Table 2:** Most significant findings.

*Neurodegenerative Diseases*		
Authors	Title	Summary
Loh J, Mak, W, Tan L, Ng C, Chan H, Yeow S, Foo, J, Ong Y, How C, Khaw K.	Microbiota–gut–brain axis and its therapeutic applications in neurodegenerative diseases.	This article investigates the gut–brain axis’s role as a regulator of glial functions and explores how it can be a therapeutic solution to neurodegenerative diseases [1].
Heravi FS, Naseri K, Hu H.	Gut microbiota composition in patients with neurodegenerative disorders (Parkinson’s and Alzheimer’s) and healthy controls: a systematic review.	According to this systematic review, patients with Alzheimer’s and Parkinson’s diseases have a distinct gut microbiota composition from healthy individuals, indicating microbial dysbiosis to be linked to the conditions [24].
Beltrán-Velasco AI, Reiriz M, Uceda S, Echeverry-Alzate V.	*Lactiplantibacillus (Lactobacillus) plantarum* as a complementary treatment to improve symptomatology in neurodegenerative disease: a systematic review of open access literature.	*Lactiplantibacillus (Lactobacillus) plantarum* has great potential for improving motor and cognitive function in persons affected by neurodegenerative diseases [64].
Bonnechère B, Amin N, van Duijn C.	What are the key gut microbiota involved in neurological diseases? A systematic review.	Gut microbiota such as Akkermansia, Faecalibacterium, and Prevotella appear altered in patients with neurological disorders such as Alzheimer’s disease and MS [68].
***Cognitive Health***		
**Authors**	**Title**	**Summary**
Handajani YS, Hengky A, Schröder-Butterfill E, Hogervorst E, Turana Y.	Probiotic supplementation improved cognitive function in cognitively impaired and healthy older adults: a systematic review of recent trials.	According to this systematic review, probiotic supplementation led to a significant improvement is the cognitive functions of older adults with Alzheimer’s disease [22].
Ticinesi A, Tana C, Nouvenne A, Prati B, Lauretani F, Meschi T.	Gut microbiota, cognitive frailty, and dementia in older individuals: a systematic review.	Alteration of the gut microbiota is linked to cognitive frailty and dementia [47].
Khine WWT, Voong ML, Ng TKS, Feng L, Rane GA, Kumar AP, Kua EH, Mahendran R, Mahendran R, Lee YK.	Mental awareness improved mild cognitive impairment and modulated gut microbiome.	Cognitive stimulation through mindful awareness helps improve cognitive impairment and induces changes in the gut microbiota, showing that brain function can influence an individual’s gut microbial profile [52].
Zhu B, Shen J, Jiang R, Jin L, Zhan G, Liu J, Sha Q, Xu R, Miao L, Yang C.	Abnormalities in gut microbiota and serum metabolites in hemodialysis patients with mild cognitive decline: a single-center observational study.	Gut microbiota and serum metabolite alterations are linked to mild cognitive decline in hemodialysis patients [69].
Kossowska M, Olejniczak S, Karbowiak M, Mosiej W, Zielińska D, Brzezicka A.	The interplay between gut microbiota and cognitive functioning in the healthy aging population: a systematic review.	Changes in the composition of the gut microbiota in healthy aging individuals can be used as markers for the onset of Alzheimer’s disease and other forms of dementia [58].
***Mental Disorders***		
**Authors**	**Title**	**Summary**
Xiong R, Li J, Cheng J, Zhou D, Wu S, Huang S, Saimaiti A, Yang Z, Gan R, Li H.	The role of the gut microbiota in anxiety, depression, and other mental disorders, as well as the protective effects of dietary components.	This study explores the role of gut microbiota in mental disorders such as anxiety and depression. Its findings show that protective dietary components can improve an individual’s mental health [38].
Arneth BM.	Gut–brain axis biochemical signaling from the gastrointestinal tract to the central nervous system: gut dysbiosis and altered brain function.	The gut–brain axis is responsible for bidirectional communication between the gut and the brain, and it plays an important role in managing mental disorders such as depression and schizophrenia [45].
Petakh P, Oksenych V, Kamyshna I, Boisak I, Lyubomirskaya K, Kamyshnyi O.	Exploring the interplay between posttraumatic stress disorder, gut microbiota, and inflammatory biomarkers: a comprehensive meta-analysis.	This meta-analysis explores the link between PTSD, immune system biomarkers, and gut microbiota. Its findings show that reduced microbial diversity could result increased immune dysregulation [70].
Olavarría-Ramírez L, Cooney-Quane J, Murphy G, McCafferty CP, Cryan JF, Dockray S.	A systematic review of the effects of gut microbiota depletion on social and anxiety-related behaviors in adult rodents: implications for translational research.	The depletion of gut microbiota among rodents shows changes in anxiety and social behaviors [42].
***Autoimmune Diseases***		
**Authors**	**Title**	**Summary**
Xu Q, Ni JJ, Han BX, Yan SS, Wei XT, Feng GJ, Zhang H, Zhang L, Li B, Pei YF.	The causal relationship between gut microbiota and autoimmune diseases: a two-sample Mendelian randomization study.	This study applies two-sample Mendelian randomization to establish a causal relationship between gut microbiota (*Bifidobacterium*) and autoimmune diseases (type 1 diabetes and celiac disease) [10].
***Cancer***		
**Authors**	**Title**	**Summary**
Li W, Zhou X, Yuan S, Wang L, Yu L, Sun J, Chen J, Xiao Q, Wan Z, Zheng JS, Zhang CX, Larsson SC, Farrington SM, Law P, Houlston RS, Tomlinson I, Ding KF, Dunlop MG, Theodoratou E, Li X.	Exploring the complex relationship between gut microbiota and risk of colorectal neoplasia using bidirectional Mendelian randomization analysis.	Genetic liability to colorectal neoplasia may affect the composition of gut microbiota, which shows a link between microbiome interactions and cancer risk [43].

## Data Availability

The data presented in this study are available on request from the corresponding author due to privacy reasons.
